# Resistance Management for Asian Citrus Psyllid, *Diaphorina citri* Kuwayama, in Florida

**DOI:** 10.3390/insects8030103

**Published:** 2017-09-20

**Authors:** Xue Dong Chen, Lukasz L. Stelinski

**Affiliations:** Citrus Research and Education Center, Department of Entomology and Nematology, University of Florida, 700 Experiment Station Rd., Lake Alfred, FL 33850, USA; xuedo1gchen@ufl.edu

**Keywords:** rotational model, resistance management strategy, leaf dip bioassay, insecticide resistance, mode of action

## Abstract

The Asian citrus psyllid, *Diaphorina citri* Kuwayma, is one of the most important pests in citrus production. The objective of this study was to evaluate *D. citri* resistance management with three insecticide rotations and compare them with no rotation and an untreated check. The different insecticides (modes of action) tested were: dimethoate, imidacloprid, diflubenzuron, abamectin 3% + thiamethoxam 13.9%, and fenpropathrin. Eggs, nymph, and adult psyllids were counted weekly. Five insecticide applications were made in 2016. Insecticide susceptibility was determined by direct comparison with a laboratory susceptible population and field populations before and after all treatments were applied. Rankings of eggs, nymphs, and adults counted in treated plots were significantly lower than in the untreated control plots after each application. Initially, the resistance ratio (RR_50_) for each rotation model, as compared with laboratory susceptible strain and the field population before application, was less than 5.76 and 4.31, respectively. However, after five applications with dimethoate, the RR_50_ using the laboratory and pre-treatment field populations was 42.34 and 34.74, respectively. Our results indicate that effectively rotating modes of action can delay and/or prevent development of insecticide resistance in populations of *D. citri*.

## 1. Introduction

The Asian citrus psyllid, *Diaphorina citri* Kuwayma, transmits a bacterium, *Candidiatus Liberibacter asiaticus* (Las), that is most likely the causal agent of citrus huanglongbing (HLB) in the U.S. HLB is an important destructive disease of citrus crops, characterized by symptoms such as leaf mottling, asymmetric chlorosis, and yellowing of veins and midribs, which resemble certain nutritional deficiencies. Infected trees produce fewer fruit that are small, lopsided, bitter-tasting, and sometimes remain green in color [1]. HLB was first detected in southern Florida in 2005 [2] and citrus production has been in decline since. The U.S. Department of Agriculture estimated 67 million boxes of orange production for 2016–2017 [3], which would be the smallest harvest in 53 years. Currently, most growers in Florida managing *D. citri* with insecticides spray on a calendar basis [4] because there is no cure for the disease and management practices still depend on insecticides to control the vector, *D. citri*. Therefore, insecticide resistance looms as a critical problem in citrus production [5,6,7].

Increasing detoxification, reduced penetration, alterations of target sites, and behavioral adaptations are common mechanisms of insecticide resistance [8,9] that may be found among *D. citri* populations. Reducing the frequency of resistance alleles within a field population is the fundamental goal of insecticide resistance management so that the efficacy of a particular insecticide can be preserved [10]. Georghiou (1983) listed three main strategies of insecticide resistance management by: (1) moderation; (2) saturation; and (3) multiple attack [11]. For the third strategy, Georghiou (1983) further subdivided into the use of insecticide mixtures and rotation of insecticides in either space or time [11]. Rotation in time has been considered both theoretically and experimentally [11,12].

Consequently, rotating different modes of action has become an established strategy for preventing decline in insecticide susceptibility [13,14,15]. Rotation should allow sufficient time between applications of any single mode of action (MOA) for resistance alleles to decline below critical frequencies when a different MOA is used [11,13,16,17]. Depending on the number of annual generations of the targeted pest, the frequency of resistant individuals within a population should decrease sufficiently during mode of action swaps when selection pressure is interrupted to prevent establishment of resistance [12,18,19]. Insecticide rotation may improve program efficacy not only by relaxing constant selection pressure to a single mode of action, but also because individuals that may be resistant to one chemistry may exhibit lower biotic fitness in the absence of that direct selection pressure than susceptible counterparts [17,20]. Therefore, insecticide rotation schemes should, in general, include as many different modes of action as possible. This is of particular importance if the mechanisms of resistance and cross resistance cannot be identified [11,21].

Insecticide resistance in field pest populations is determined by several factors associated with the target species, including genetic, biological, environmental, ecological, chemical and operational such as application timing [9,22]. Application timing and sequence can be controlled to alter the specific selection stress by which resistance develops. A spectrum of approaches for timing of insecticide applications has been proposed, such as sequential use of a chemical until resistance develops versus regularly alternating different modes of action [11]. The tactic of using rotations of insecticides was proposed on the assumption that resistance to the first insecticide would decline during the application of an alternate [13]. Although tactics for managing resistance to insecticides are well known and have been discussed extensively [13,19,23], optimizing a strategy for preventing or delaying resistance for a specific target requires field verification. The objective of this study was to evaluate various insecticide rotations for management of *D. citri* in Florida. The purpose of the investigation was not to measure field efficacy of programs (although such information was measured), but to specifically determine if specific rotations could delay resistance development in field populations of *D. citri*. Various modes of action used in Florida citrus pest management were evaluated in three distinct sequences of application as compared with both constant and no selection pressure.

## 2. Materials and Methods

### 2.1. Insect Rearing and Chemicals

A susceptible laboratory population of *D. citri* was reared in a greenhouse at the Citrus Research and Education Center, University of Florida, Lake Alfred, Florida. It originated from adults collected in 2000 from citrus in Polk County, FL. This strain has been reared without exposure to insecticides. The culture was maintained on sweet orange (*Citrus sinensis* (L) Osbeck) in a greenhouse at about 27 °C, with 60% relative humidity and a 14:10 (light: dark) photoperiod. Field populations were collected from a 2.01 ha sweet orange grove (cv. ‘Hamlin’) in Winter Garden, Florida (N: 28°27′965″; W: 81°39′542″) before and after application of three different insecticide rotation schemes. Also, sequential applications of a single mode of action without rotation and an untreated control treatment were used as comparisons. Tested insecticides were commercial formulations. The trade names, insecticide active ingredients, Insecticide Resistance Actions Committee (IRAC) classifications, manufacturers, application rates, application methods, and modes of action are listed in Table 1.

### 2.2. Insecticide Application 

Plots were established in a sweet orange grove cv. ‘Hamlin’ in Winter Garden, Florida. Our site had not been treated with insecticides for the prior three years. The plots were 11 × 23 m in size with an 8.2 m buffer between plots and 13.7 m spacing between blocks. The plots consisted of five rows of four trees per row. A total of 20 trees were sampled within each plot, which were chosen at random from the central second and third rows. The design was a randomized complete block with four replicates. This design was space limited in scale and assumed >90% of psyllids moved <7 m during their lifetime as documented by Kobori et al. (2011) using mark–release–recapture investigations [24]. The rotational schemes are shown in Table 2. The plots were sampled weekly beginning on 17 March 2016. The tap sample method of Hall & Hentz (2010) was used to sample adults [25]. Ten tap samples were taken per plot. For eggs and nymphs, 10 randomly selected flush samples were taken per pot and the number of eggs and nymphs per flush were quantified by a previously established ranking method [26]. The rankings for eggs were: 0 = 0; 1 = 1–20; 2 = 21–40; 3 > 41, and for nymphs were: 0 = 0; 1 = 1–5; 2 = 6–10; 3 > 11. Applications of insecticides were made based on psyllid populations reaching a predetermined threshold. The predetermined thresholds were: 1.2 adults per tap sample per 10 samples per plot, egg ranking >1 per flush sample or nymph ranking >1.5 per flush sample on average per plot. Beginning on 17 March 2016 and weekly thereafter, the plots were sampled to estimate infestation levels of eggs, nymphs, and adults. Applications were made on 24 May, 23 June, 27 July, 7 September, and 5 October 2016 with a handgun. Trees were sprayed until runoff. The treatments (Table 2) were evaluated by weekly ranks of *D. citri* egg, nymph, and adult tap counts.

### 2.3. Leaf Dip Bioassay to Determine Insecticide Susceptibility 

The treatments evaluated were the three rotations, a positive control, and one negative (untreated) control as described above. The overall experiment was initiated with an existing field population of *D. citri,* which was sampled initially prior to the experiment on 11 April 2016. For each insecticide, mortality was compared with the laboratory susceptible strain, and within each treatment population with psyllids collected in April 2017 to generate resistance ratios as described below in the statistical analysis section.

The changes in susceptibility of adult *D. citri* populations to insecticides before and after the final field applications were determined by the leaf dip bioassay as described in Prabahker et al. (2006) [27] and Boina et al. (2009) [28]. In brief summary, commercial formulations of dimethoate, fenpropathrin, imidacloprid, and abamectin + thiamethoxam mixture were investigated. Five to six concentrations of each insecticide were tested in the laboratory bioassay and replicated 4–5 times. Each insecticide was diluted in distilled water. Citrus leaves were collected from ‘Valencia’ orange trees that were not treated with insecticide and 35 mm diameter leaf discs were cut. The leaf discs were dipped in test solutions for 30 s and were allowed to dry in a fume hood for 1 h. Leaf discs dipped in water alone served as controls. Each concentration of each insecticide was replicated four to five times. Thereafter, 35 mm Petri dishes (Thermo Fisher Scientific, Waltham, MA, USA) were coated with a 1.5% agar solution to form a bed after solidification to maintain the integrity of each leaf disc. In total, 277–350 individuals were tested on each sampling date for each insecticide within each rotational or control treatment using the leaf dip bioassay. *D. citri* were anesthetized briefly with CO_2_ and 10 adults were placed into each Petri dish.

Mortality counts were taken 48 h after transfer into a growth chamber at the environmental conditions described above for insect rearing. Insects found on their side or back that were unable to move upon prodding were considered dead and included in mortality counts.

### 2.4. Statistical Analysis

The number of adults counted was analyzed using two-way mixed model analysis of variance (ANOVA) (treatment × replication). The first order interaction was removed from the analytical model if it was not significant [29]. If the first order interaction was significant, differences between treatments were determined with the Bonferroni test. Rankings of eggs and nymphs were analyzed using the Kruskal–Wallis *H*-Test (*p* = 0.05).

Mortality data were analyzed by probit analysis [30] and were computed using SAS statistical software PROC probit SAS 9.4 [31]. A likelihood ratio test was conducted to test the hypothesis that all *p* values were equal. The mortality of the control treatment never exceeded 3% and Abbott’s formula was used to adjust for mortality of the control when it occurred [32].

Resistance ratios (RRs) were determined using the laboratory susceptible population as a baseline and the field population prior to insecticide application as a second baseline. The RR_50_ for the laboratory baseline was calculated by dividing the LC_50_ of the field population after insecticide applications were made by the LC_50_ of the laboratory population. The RR_50_ for the field baseline was calculated by dividing the LC_50_ of the field population after the applications by the LC_50_ of the field population before insecticide applications were made. RR_95_ was calculated as described above using the appropriate LC_95_. Different resistance levels were categorized based on the following scale: Resistance ratio (RR) = 1, no resistance; RR = 2–10, very low resistance; RR = 11–20, low resistance; RR = 21–50, moderate resistance; RR = 51–100, high resistance; and RR > 100 very high resistance [6,33,34,35,36,37]. All raw data used in statistical analyses can be found in the Supplementary Materials.

## 3. Results

### 3.1. Monitoring of Egg, Nymph, and Adult Psyllids to Determine Thresholds for Application 

On 17 March, there were no significant differences between treatments for egg ranking (χ^2^ = 6.39; *df* = 0.17; *p* = 0.17); however, there was a significant difference for nymph ranking (χ^2^ = 29.54; *df* = 4; *p* < 0.0001) (Table 3). There were no significant differences between replicates (χ^2^ = 6.2; *df* = 3; *p* = 0.10). On this sampling date, there was no significant treatment × replication interaction for adult psyllid counts (*F* = 0.89; *df* = 7; *p* = 0.52) (Table 3).

On 19 May, there was a significant difference in egg ranking between treatments (χ^2^ = 13.98; *df* = 4; *p* = 0.007) (Table 3), but no significant difference between replicates (χ^2^ = 4.60; *df* = 3; *p* = 0.20). Nymph rankings also differed significantly between treatments (χ^2^ = 13.98; *df* = 4; *p* = 0.0074) (Table 3), with no significant differences between replicates (χ^2^ = 3.37; *df* = 3; *p* = 0.34). For adult counts, there was no significant treatment × replication interaction (*F* = 1.33; *df* = 5; *p* = 0.26) (Table 3). On this date (19 May), our predetermined spray threshold was reached and we initiated the first treatment application according to the rotation schedules given in Table 2. All subsequent applications were based on psyllid populations reaching the predetermined threshold described previously. Given that populations of *D. citri* increased at different rates throughout the season, depending on host phenology, spray applications occurred at irregular intervals over the course of the investigation when the threshold was triggered.

### 3.2. Monitoring of *D. citri* Eggs, Nymphs, and Adults after Application

After the first application, we monitored egg, nymph and adult stages weekly in each treatment. After the 24 May application, ranks of *D. citri* eggs and nymphs, as well as, the number of adults counted were significantly lower in plots treated with each of the three rotation treatments or the sequential dimethoate treatment positive control as compared with untreated negative control (*p* = 0.05). The average ranking for *D. citri* nymphs following five sequential applications of dimethoate was significantly higher than following five applications of rotated insecticides for each of the three rotation treatments tested (*p* < 0.05) (Table 4).

### 3.3. Insecticide Resistance

The RR_50_ values are given in Table 5, Table 6, Table 7 and Table 8. Only insects in plots treated with dimethoate without rotation showed moderate levels of resistance to this chemical (Table 7). Resistance was not detected to any of the insecticides tested in any of the rotation treatments (Table 5, Table 6, Table 7 and Table 8).

## 4. Discussion

In Florida, instances of susceptibility shifts of *D. citri* populations have been documented for several insecticides [5,6]. A common method of managing resistance is to rotate modes of action within annual insecticide spray programs [17,38]. This is of particular importance for sub-tropical pests, such as *D. citri*, which occurs year round and can require 12 or more annual management sprays [4]. Herein, we compared various rotation programs to determine whether we could delay or prevent development of resistance in populations of *D. citri* in Florida citrus.

Each of the three rotation models evaluated effectively prevented development of resistance in populations of *D. citri*. Evidence of reduced susceptibility to dimethoate occurred only after five sequential applications of this insecticide in the positive control, no rotation plots. The resistance ratios (RR_50_) for *D. citri* collected from these sequentially treated plots increased from 5.76 and 4.31 to 42.34 and 34.74, when compared against the laboratory susceptible population and the field population prior to the initial application, respectively. However, *D. citri* collected from field plots where dimethoate was rotated with four other modes of action and never applied multiple times in sequence (irrespective of application order) showed no signs of decreased susceptibility to dimethoate. These results are congruent with other field-based investigations with other insects. Immaraju et al. (1990) reported that insecticide rotation was a superior to insecticide mixtures for resistance management of citrus thrips, *Scirtothrips citri* (Moulton) [14]; however, both rotation and mixtures were better than sequential use. Zahiri et al. (2002) showed that treatment rotations reversed existing resistance among populations of *Bacillus sphaericus* Neide [39]. Jonsson et al. (2010) demonstrated that rotation between spinosad and amitraz successfully suppressed a *Rhipicephalus* (*Boophilus*) *microplus* population with amitraz resistance [40]. Furthermore, McKenzie et al. (2014) showed effective management of *Bemisia tabaci* (Gennadius) on poinsettia while mitigating development of resistance by rotating applications of chemical classes [41].

Although not recommended and often avoided, sequential application of the same insecticide mode of action has occurred in Florida citrus (LS personal observation). It should therefore not be surprising that insecticide resistance among populations of *D. citri* was documented in Florida as early as 2009 [42,43] and continues to be a problem [5]. In other regions of the world, the magnitude (4000-fold) of insecticide resistance to MOAs such as organophosphates documented for populations of *D. citri* should predict failures of applied products in the field [44]. Although we did not conduct direct yield estimates to evaluate treatment efficacy because the impact on insecticide susceptibility of *D. citri* was the focus of the investigation, there did not appear to be differences between the rotation modules and the overall number of *D. citri* found in plots over the course of the investigation. Larger scale investigation over multiple seasons would be necessary to verify whether any of these particular rotation schedules is more efficacious for management of *D. citri* than the others tested and to determine the underlying mechanism(s). It is unclear from this investigation whether the decrease in organophosphate susceptibility of *D. citri* in plots treated repeatedly with dimethoate caused reduced efficacy of insecticide sprays in these plots gradually over time. Also, the small scale of the experiment did not preclude movement of adult *D. citri* between plots and thus interbreeding likely diluted population-wide effects of the differing selection pressures. However, our investigation demonstrated the value of implementing MOA rotation to manage resistance for *D. citri*, despite its small-scale design. These results may have been even more marked if conducted on a larger scale to prevent the local population from inbreeding between treatment applications. Rotation of insecticide modes of action does prevent or delay onset of resistance in *D. citri* populations, and the effects of even relatively short-duration selection pressure can be observed even in small plots where inbreeding with non-selected psyllids is possible. Further evaluations of rotations for *D. citri* insecticide resistance and vector management are warranted on a larger scale and among a greater regional diversity of *D. citri* populations in Florida because field populations vary widely in their current susceptibility to labeled insecticides [5,42]. Comparing more field populations and on a larger scale may reveal efficacy differences between sequences of rotated MOAs. Also, determining duration required for reversal to susceptibility is needed in future investigations in cases where populations resistant to a certain MOAs occur at test onset.

## 5. Conclusions

Our study demonstrated that continuous selection pressure on a highly localized population of *D. citri* with a single MOA can cause 30–40-fold reduction in susceptibility to an organophosphate (dimethoate) after only five sequential applications under field conditions. Also, higher counts of *D. citri* nymphs in plots treated sequentially five times with dimethoate as compared with the rotation treatments suggests this decrease in insecticide susceptibility may have been associated with decreased product efficacy. Our results indicate that failure to rotate MOAs during *D. citri* management with insecticides in Florida citrus production may establish localized populations of insecticide resistant *D. citri*.

## Figures and Tables

**Table 1:** Formulated insecticides evaluated as part of rotation modules for management of Asian citrus psyllid, *Diaphorina citri*.

TRADE NAME	Active Ingredients	IRAC ^a^	Rate/Acre (lb)	Chemical Class	MOA ^b^	Manufacturer
Dimethoate 4E	Dimethoate (43.5%)	1B	1.0	Organophosphate	AChE inhibitor	Cheminova
Admire Pro 4.6F	Imidacloprid (42.8%)	4A	0.5	Neonicotinoid	nAChR competitive modulator	Bayer Crop Science
Micromite 80WGS ^c^	Diflubenzuron (80%)	15	0.39	Insect growth regulator	Inhibitor of chitin biosynthesis	Chemtura
Agri-Flex ^c^	Abamectin 3% + Thiamethoxam 13.9%	6 + 4A	0.53	Microbial + Neonicotinoid	GluCl allosteric + nAChR competitive modulator	Syngenta Crop Protection
Danitol 2.4EC	Fenpropathrin (30.9%)	3A	0.40	Pyrethroid	Sodium channel modulator	Valent Inc.

^a^ IRAC: Insecticide Resistance Action Committee (IRAC) classification; ^b^ MOA = mode of action; AChE: Acetylcholinesterase; nAChR: Nicotinic acetylcholine receptor; GluCI: Glutamate gated chloride channels; ^c^ with IAP 435 CROP oil 0.2% *v*/*v*.

**Table 2:** Rotation schedule treatments and dates of application.

Treatment	Date of Application				
	24 May 2016	23 June 2016	27 July 2016	7 September 2016	5 October 2016
**Rotation A**	dimethoate	abamectin + thiamethoxam	fenpropathrin	diflubenzuron	imidacloprid
**Rotation B**	imidacloprid	fenpropathrin	abamectin + thiamethoxam	dimethoate	diflubenzuron
**Rotation C**	abamectin + thiamethoxam	diflubenzuron	dimethoate	imidacloprid	fenpropathrin
**Sequential dimethoate**	dimethoate	dimethoate	dimethoate	dimethoate	dimethoate
**Untreated control**	-	-	-	-	-

**Table 3:** Average density rankings (±SE) of Asian citrus psyllid, *Diaphorina citri*, egg, nymph, and adult counts upon plot establishment and immediately prior to first treatment application.

Rotation Program	17 March 2016 ^a^			19 May 2016 ^b^		
	Egg	Nymph	Adult	Egg	Nymph	Adult
**Rotation A**	0.05 ± 0.05	0.03 ± 0.02	0.00 ± 0.00	1.10 ± 0.21	1.81 ± 0.20	1.50 ± 0.27
**Rotation B**	0.08 ± 0.04	0.27 ± 0.07	0.05 ± 0.04	1.43 ± 0.16	2.43 ± 0.17	1.18 ± 0.28
**Rotation C**	0.10 ± 0.05	0.33 ± 0.11	0.08 ± 0.04	1.24 ± 0.18	2.15 ± 0.19	1.03 ± 0.20
**Sequential dimethoate**	0.03 ± 0.03	0.11 ± 0.07	0.05 ± 0.05	1.69 ± 0.18	2.51 ± 0.13	1.25 ± 0.28
**Untreated control**	0.28 ± 0.16	0.67 ± 0.13	0.00 ± 0.00	1.06 ± 0.20	1.61 ± 0.23	1.64 ± 0.30

^a^ Monitoring initiated; ^b^ Above threshold prior to first application.

**Table 4:** Average density ranking (±SE) of Asian citrus psyllid, *Diaphorina citri*, egg, nymph, and adult counts during rotation treatments (31 May 2016 to 26 October 2016).

Rotation Program	Egg (Mean ± SE)	Nymph (Mean ± SE)	Adult (Mean ± SE)
**Rotation A**	0.69 ± 0.03a	0.89 ± 0.04a	0.19 ± 0.03a
**Rotation B**	0.73 ± 0.03a	1.02 ± 0.04a	0.31 ± 0.03a
**Rotation C**	0.73 ± 0.03a	1.05 ± 0.04a	0.13 ± 0.03a
**Sequential dimethoate**	0.62 ± 0.03a	1.23 ± 0.04b	0.23 ± 0.03a
**Untreated control**	0.97 ± 0.03b	1.67 ± 0.04c	0.83 ± 0.03b

Mean number of eggs, nymphs, and adults followed by same letter in the columns are not significant (Bonferroni test; *p* < 0.05).

**Table 5:** Susceptibility of *Diaphorina citri* adults exposed to fenpropathrin from field plots under different treatment rotations.

Population	N	χ^2^	Slope ± SE	LC_50_ (ng/µL) ^a^ 95% FL	LC_95_(ng/µL) (95% FL) ^b^	L-RR_50_	F-RR_50_	L-RR_95_	F-RR_95_
**Lab strain**	350	4.55	1.20 ± 0.13	0.15 (0.09–0.23)	3.42 (1.70–9.3)	1		1	
**Field strain before application**	300	6.01	0.87 ± 0.10	0.16 (0.09–0.27)	12.14 (5.13–43.03)	1.07	1	3.55	1
**Rotation A**	288	96	1.00 ± 0.16	0.31 (0.09–1.15)	13.44 (2.8–584.2)	2.07	1.93	3.93	1.11
**Rotation B**	292	8.06	0.93 ± 0.14	0.23 (0.07–0.78)	13.28 (2.8–363.6)	1.53	1.43	3.88	1.09
**Rotation C**	289	3.84	1.14 ± 0.13	0.22 (0.13–0.36)	6.09 (2.86–18.52)	1.47	1.38	1.78	0.50
**Sequential dimethoate**	292	9.09	0.93 ± 0.14	0.24 (0.07–0.80)	13.59 (2.9–360.4)	1.60	1.50	4.00	1.12
**Untreated control**	293	6.42	1.06 ± 0.12	0.23 (0.14–0.38)	8.08 (3.67–25.39)	1.53	1.44	2.36	0.67

^a^ L-RR_50_ and L-RR_95_ = Field LC_50_ or Field LC_95_/Lab LC_50_ or Lab LC_95_; F-RR_50_ or F-RR_95_ = after application LC_50_ or LC_95_/before application LC_50_ or LC_95_; ^b^ FL = Fiducial Limits.

**Table 6:** Susceptibility of *Diaphorina citri* adults exposed to abamectin 3% + thiamethoxam 13.9% from field plots under different treatment rotations.

Population	N	χ^2^	Slope ± SE	LC_50_ (ng/µL) ^a^ 95% FL	LC_95_(ng/µL) (95% FL) ^b^	L-RR_50_	F-RR_50_	L-RR_95_	F-RR_95_
**Lab strain**	300	1.11	0.76 ± 0.09	0.09 (0.05–0.17)	13.64 (2.28–56.84)	1		1	
**Field strain before application**	350	1.77	0.74 ± 0.13	0.12 (0.06–0.22)	19.41 (7.62–75.97)	1.33	1	1.42	1
**Rotation A**	285	5.70	0.90 ± 0.10	0.04 (0.02–0.07)	2.83 (1.16–10.47)	0.44	0.33	0.21	0.15
**Rotation B**	281	7.33	0.79 ± 0.09	0.05 (0.01–0.16)	4.65 (1.72–20.06)	0.56	0.42	0.34	0.24
**Rotation C**	284	2.49	0.87 ± 0.10	0.04 (0.02–0.07)	2.78 (1.11–10.65)	0.44	0.33	0.20	0.14
**Sequential dimethoate**	283	2.91	0.95 ± 0.11	0.05 (0.03–0.08)	2.37 (1.01–8.20)	0.56	0.42	0.17	0.12
**Untreated control**	283	8.85	0.96 ± 0.16	0.07 (0.02–0.26)	3.76 (0.78–135.1)	0.78	0.58	0.27	0.19

^a^ L-RR_50_ and L-RR_95_ = Field LC_50_ or Field LC_95_/Lab LC_50_ or Lab LC_95_; F-RR_50_ or F-RR_95_ = after application LC_50_ or LC_95_/before application LC_50_ or LC_95_; ^b^ FL = Fiducial Limits.

**Table 7:** Susceptibility of *Diaphorina citri* adults exposed to dimethoate from field plots under different treatment rotations.

Population	N	χ^2^	Slope ± SE	LC_50_ (ng/µL) ^a^ 95% FL	LC_95_(ng/µL) (95% FL) ^b^	L-RR_50_	F-RR_50_	L-RR_95_	F-RR_95_
**Lab strain**	300	5.81	1.04 ± 0.11	0.32 (0.19–0.51)	11.80 (5.56–34.94)	1		1	
**Field strain before application**	300	5.92	1.14 ± 0.12	0.39 (0.24–0.66)	10.51 (4.80–34.18)	1.22	1	0.89	1
**Rotation A**	277	7.49	0.81 ± 0.09	1.68 (0.91–3.22)	172.92 (60.9–789.1)	5.25	4.31	14.65	16.5
**Rotation B**	283	13.81	0.93 ± 0.18	0.39 (0.07–2.30)	23.20 (3.48–3877)	1.22	1	1.97	2.21
**Rotation C**	277	19.47	0.90 ± 0.21	1.20 (0.14–16.59)	79.98 (7.97–554,715)	3.75	3.08	6.78	7.61
**Sequential dimethoate**	283	14.73	0.56 ± 0.14	13.55 (1.45–3694)	11,126 -	42.34	34.74	952.88	1058
**Untreated control**	290	4.30	0.75 ± 0.08	0.48 (0.10–20.57)	73.69 (26.1–321.3)	1.5	1.23	6.25	7.01

^a^ L-RR_50_ and L-RR_95_ = Field LC_50_ or Field LC_95_/Lab LC_50_ or Lab LC_95_; F-RR_50_ or F-RR_95_ = after application LC_50_ or LC_95_/before application LC_50_ or LC_95_; ^b^ FL = Fiducial Limits.

**Table 8:** Susceptibility of *Diaphorina citri* adults exposed to imidacloprid from field plots under different treatment rotations.

Population	N	χ^2^	Slope ± SE	LC_50_ (ng/µL) ^a^ 95% FL	LC_95_(ng/µL) (95% FL) ^b^	L-RR_50_	F-RR_50_	L-RR_95_	F-RR_95_
**Lab strain**	350	9.42	0.32 ± 0.14	0.33 (0.06–1.30)	95.01 (13.79–7383)	1		1	
**Field strain before application**	350	9.04	0.10 ± 0.15	0.75 (0.09–3.83)	385.98 -	2.27	1	4.06	1
**Rotation A**	280	1.31	0.44 ± 0.06	1.90 (0.75–5.64)	10,390 -	5.76	2.53	109.3	26.92
**Rotation B**	281	1.99	0.57 ± 0.06	0.71 (0.33–6.60)	576.40 (135–5023)	2.15	0.94	6.06	1.49
**Rotation C**	280	1.69	0.56 ± 0.07	1.17 (0.54–2.67)	1024 -	3.54	1.56	10.77	2.65
**Sequential dimethoate**	280	1.70	0.46 ± 0.06	1.63 (0.67–4.54)	5664 -	4.94	2.17	59.61	14.67
**Untreated control**	280	3.62	0.54 ± 0.06	0.81 (0.37–1.87)	878.77 (187–9159)	2.46	1.08	9.24	2.28

^a^ L-RR_50_ and L-RR_95_ = Field LC_50_ or Field LC_95_/Lab LC_50_ or Lab LC_95_; F-RR_50_ or F-RR_95_ = after application LC_50_ or LC_95_/before application LC_50_ or LC_95_; ^b^ FL = Fiducial Limits.

## Supplementary Materials

The following are available online at www.mdpi.com/2075-4450/8/3/103/s1.
